# Who are the healthy active seniors? A cluster analysis

**DOI:** 10.1186/1471-2318-14-127

**Published:** 2014-12-01

**Authors:** Claudia K Y Lai, Engle Angela Chan, Kenny C W Chin

**Affiliations:** School of Nursing (SN), The Hong Kong Polytechnic University (PolyU), Hung Hom, Kowloon, Hong Kong Special Administrative Region (HKSAR), Kragujevac, China; SN, PolyU, Hung Hom, Kowloon, HKSAR, Kragujevac, China

**Keywords:** Ageing, Elderly, Community, Cluster analysis

## Abstract

**Background:**

This paper reports a cluster analysis of a sample recruited from a randomized controlled trial that explored the effect of using a life story work approach to improve the psychological outcomes of older people in the community.

**Methods:**

238 subjects from community centers were included in this analysis. After statistical testing, 169 seniors were assigned to the active ageing (AG) cluster and 69 to the inactive ageing (IG) cluster.

**Results:**

Those in the AG were younger and healthier, with fewer chronic diseases and fewer depressive symptoms than those in the IG. They were more satisfied with their lives, and had higher self-esteem. They met with their family members more frequently, they engaged in more leisure activities and were more likely to have the ability to move freely.

**Conclusion:**

In summary, active ageing was observed in people with better health and functional performance. Our results echoed the limited findings reported in the literature.

## Background

Active ageing is discussed in the literature as a goal that health professionals, policy makers, and the general public would like to attain. The term ‘active ageing’ was first adopted by the World Health Organization (WHO) in the late 1990s [1]. The WHO defined active ageing as the process of optimizing opportunities for health, participation, and security in order to enhance quality of life as people aged (p. 12) [2]. To date, there is no universally accepted definition of ‘active ageing’ [3]. The concepts of active ageing overlap with those of healthy ageing, productive ageing, or successful ageing – some terms that commonly appear in the literature.

Healthy ageing as a concept has been defined in various ways and with different underlying assumptions [4]. In general, the concept of healthy ageing has been described as a complex process of adapting to physical and socio-psychological changes over one’s lifetime [5]. Healthy ageing is not clearly defined as a concept in many published reports and papers, but ideas surrounding the concept are discussed with the assumption that individuals who age healthily are those who are more independent in daily activities and free from debilitating illnesses.

Rowe and Kahn defined successful aging not only as achieving better outcomes of physical and cognitive health, but also being actively engaged with life [6]. Bowling and Dieppe conducted a systematic review on the models of successful ageing and concluded that there are two main approaches to understanding the concept – the psychosocial school, which defines successful ageing as wellbeing of the mental states such as life satisfaction, and the biomedical school, which interprets the concept as the prevention of diseases and disabilities [7]. A consensus has yet to be reached on the definition of successful ageing [8]. Still, success in ageing is commonly defined as how well an older person has maintained or achieved better health outcomes.

The concept of productive ageing conceptualizes ageing from a somewhat different perspective. The productive ageing framework transcends the physical or functional realm of an individual’s health and views an older person who is capable of accomplishing his/her goals and tasks as having a sense of purpose in life [9]. The viewpoint focuses on the older adults’ contributions to society as well as their internal affective state, which may have a positive impact on their wellbeing. Both the external and internal (to the older person) views emphasize engagement by older adults in their physical, psychological, and socioeconomic environment.

In summary, there are some common concepts in these terms relating to ageing, but in conceptualization they differ somewhat in focus. The definitions of various terms are arbitrarily used in a variety of contexts [5]. Gerontologists – whether clinicians or researchers – have not come any closer to reaching a consensus after years of deliberation.

As a multidimensional concept, the term active ageing includes concepts of activity, health, independence, and productivity in older age [10, 11]. The term is intended to convey a more inclusive message than ‘healthy ageing’ and to recognize factors in addition to health care that affect how individuals and populations age [1]. Older persons with chronic disease may still be considered healthy if they are socially and intellectually active [12]. Despite keen discussions of the concept of active ageing, there are few reports in the literature on the determinants of active ageing or the factors associated with it.

A systematic search of the literature using the search engine EBSCOHost to access the databases CINAHL, MEDLINE (1965+), and Social Work Abstracts was conducted. The following keywords were used without choosing any fields to search for relevant papers for the period 1978 to 2013, on the determinants of or factors associated with active ageing - (“active ageing” OR “active aging”) AND (“determinants” OR “factors” OR “associat*”). Eighteen papers were found and 13 papers remained after duplicates were culled.

Among these 13 papers, three did not focus on active ageing although the term was mentioned [13–15]; another three contained conceptual discussions of the term [16–18]; two were about older people’s perceptions of active ageing [19, 20]; four were about other concepts related to ageing or active ageing – e.g., the concept of quality of life [21], successful ageing [22], and how neighborhood influenced function [23]; and one study reported using focus group interviews to examine whether an education programmed for active ageing would lead to participant empowerment after training [24]. Only one – Eskurza et al. studied the role of oxidative stress in physically active ageing in humans [25]. Although not an exhaustive search of all health-related databases, our search showed that there is a dearth of studies on the determinants of active ageing or the factors associated with it.

We conducted a randomized controlled trial (RCT) to determine whether the production of a life story book (LSB) as an intervention for community-dwelling subjects would lead to better psychological outcomes (referring to a higher level of life satisfaction, better self-esteem, and improved well-being). In the course of our study, the project team observed that there seemed to be some differences in outcomes between older adults who were physically and functionally active as opposed to those who were not. We therefore proceeded to conduct a systematic literature review (as discussed above) and a cluster analysis to examine the validity of our clinical impression. This paper reports the results of a cluster analysis of the data generated from this sample. The objective of this report was to identify the profile of active older people in this community study in relation to their psychosocial status. Ethical approval was obtained from the University’s Human Subjects Ethics Application Review Committee (synonymous with an ethics review board in the West). Informed written consent was obtained from all of the subjects.

## Methods

The project team collaborated with a non-profit-making non-governmental organization in Hong Kong. Its elderly services section runs 17 community and day care centers for subjects serving the local population.

### Sampling and the sample

All active members of the 17 centers were used as the sampling frame. A random sample of 244 subjects who (i) were aged 60 or above, (ii) were able to understand and speak Cantonese, (iii) were able to see and hear with or without aids, and (iv) did not have any active major illnesses or psychosocial crises, were recruited and randomly assigned into an intervention and a control group.

### Intervention and control conditions

The intervention was the construction of an individual LSB using a life story approach developed by the first author. It was a one-to-one intervention consisting of four to six weekly sessions lasting from 30 to 60 minutes per session. The control condition consisted of social activities unrelated to the discussion of the subjects’ own life stories.

### Data collection

The demographic and clinical characteristics of the participants, including gender, age, date of birth, education, presence of chronic illness, number of medical diagnoses, medications, income source and level, dwelling status, types of leisure activities engaged in, exercise pattern, and presence of sleep problems were collected. Other clinical and control variables that were collected included hearing and vision, the Modified Barthel Index and the Instrumental Activities of Daily Living scale for assessing the participants’ functional performance, and the Lubben Social Network Scale and Life Events scale to control for the presence of any psychosocial variables that might have an impact on the outcomes.

The outcome measures included the Life Satisfaction Scale Index A (LSI-A), Rosenberg’s Self-Esteem Scale (RSES), the General Health Questionnaire (GHQ), and the Geriatric Depression Scale (GDS), which were collected at baseline (T0), immediately post-intervention (T1), 3 months post-intervention (T2), and 6 months post-intervention (T3). We have also collected information about the subjects’ leisure activities and exercise patterns. The baseline data at T0 were used for data analysis because the intervention and the control conditions are induced activities. All of these measures have been validated for use in Hong Kong by local researchers, and with good psychometric properties.

### Data analysis

Of the 244 randomized subjects, 238 were included in the analysis because of missing data in the GDS, GHQ, LSI-A and RSES scores of the rest of the cases. A two-step clustering approach, namely agglomerative hierarchical cluster analysis and k-means cluster analysis, was used to develop a system of typology for characterizing factors that contribute to those subjects who were more satisfied with their lives and had higher self-esteem, and who were healthier and less depressed. In addition, discriminant analysis was used to develop a screening model that would allow us to allocate the subjects into an appropriate grouping based on the range of the characteristics to be tested.

Measures including GDS, GHQ, LSI-A, and RSES, which were our primary outcomes, were used to determine to which clusters the subjects belonged. Agglomerative hierarchical cluster analysis was first used to decide on the optimal number of clusters among the subjects, which was based on statistics, namely semi-partial R-squared (SPRSQ), Cubic clustering criteria (CCC), and pseudo F (PSF). These statistics provide information about the cluster solution at any given step (i.e., the new cluster that formed at this step, and the consequences of forming the new cluster). The value with a large percentage decrease in SPRSQ at a given cluster refers to that cluster number as the optimal cluster solution, while large values for the CCC and PSF at a given cluster suggest a good stopping point for the cluster solution. The results in Table 1 show that the CCC and the PSF have the highest values at cluster 2. A large decrease in SPRSQ is also detectable in that cluster, implying that cluster 2 is the best solution.

**Table 1:** **SPRSQ, CCC & PSF values at given cluster(s)**

Cluster	SPRSQ	CCC	PSF
10	0.0146	8.13	85.6
9	0.0154	8.28	88.8
8	0.0201	8.2	91.7
7	0.0203	8.67	97
6	0.0257	9.2	103
5	0.0395	9.15	109
4	0.0437	14.4	120
3	0.0531	21.3	146
2	0.1463	23.7	162
1	0.4065	0	

Note : SPRSQ = Semi-partial R-squared,

CCC = Cubic clustering criteria,

PSF = Pseudo F.

## Results

After determining the number of the clusters, k-means clustering was then used to allocate the seniors into an appropriate cluster. As a result, two clusters were formed and named active ageing (AG) and inactive ageing (IG). One hundred and sixty-nine seniors were assigned to AG and 69 to IG. Their profile is presented in Table 2.

**Table 2:** **Characteristics of the active and inactive clusters**

		Active cluster		Inactive cluster			
Profile characteristics		(N =169)		(N = 69)		Chi-square test	
		N	%	N	%	χ2	p-value
Gender						0.098	.755
Male		48	28.4%	21	30.4%		
Female		121	71.6%	48	69.6%		
Marital status						1.276	.735
Married		72	42.6%	26	37.7%		
Widow		82	48.5%	34	49.3%		
Separate		4	2.4%	3	4.3%		
Single		11	6.5%	6	8.7%		
Main income source						24.932	.000
From government		50	30.1%	45	65.2%		
From family members		101	60.8%	21	30.4%		
Own savings		15	9.0%	3	4.3%		
Monthly income						13.161	.041
Less than 2,000		23	15.1%	20	30.8%		
2,000 - 3,999		76	50.0%	36	55.4%		
4,000 – 5,999		33	21.7%	6	9.2%		
6,000 – 7,999		6	3.9%	1	1.5%		
8,000 – 9,999		7	4.6%	1	1.5%		
10,000 – 14,999		5	3.3%	1	1.5%		
15,000 – 19,999		2	1.3%	0	.0%		
Enough money for daily expense						16.172	.003
Extremely insufficient		4	2.4%	4	5.9%		
Not enough		16	9.5%	15	22.1%		
Just enough		78	46.4%	36	52.9%		
Enough		61	36.3%	13	19.1%		
In excess		9	5.4%	0	.0%		
Living status						8.092	.088
Alone		60	35.5%	37	53.6%		
Live with spouse		38	22.5%	13	18.8%		
Live with spouse & children		23	13.6%	5	7.2%		
Live with children		39	23.1%	13	18.8%		
Live with other people		9	5.3%	1	1.4%		
Sport habit						2.322	.508
Less than one day per week		15	9.0%	9	13.4%		
1 - 3 days per week		33	19.9%	17	25.4%		
4 - 5 days per week		18	10.8%	7	10.4%		
6 - 7 days per week		100	60.2%	34	50.7%		
Sleepless night						18.914	.000
Less than one day per week		119	71.7%	28	41.8%		
1 - 3 days per week		30	18.1%	22	32.8%		
4 - 5 days per week		5	3.0%	5	7.5%		
6 - 7 days per week		12	7.2%	12	17.9%		
Leisure activities engaged in*							
Mahjong		33	19.8%	6	9.1%	1.966	0.049
Reading/writing		57	34.1%	11	16.7%	2.642	0.008
Sing/dancing/musical instrument		44	26.3%	6	9.1%	2.891	0.004
Play chess		11	6.6%	2	3.0%	1.066	0.287
Watching TV/listen to radio		102	61.1%	52	78.8%	-2.573	0.010
Hearing						1.424	.491
Normal		121	71.6%	44	63.8%		
Slightly difficult		37	21.9%	19	27.5%		
Fairly difficult		11	6.5%	6	8.7%		
Difficult		0	.0%	0	.0%		
Vision						14.757	.002
Fine		141	83.4%	43	62.3%		
Slight problem		22	13.0%	16	23.2%		
Mild problem		5	3.0%	8	11.6%		
Moderately difficult		1	.6%	2	2.9%		
Difficult		0	.0%	0	.0%		
Climbing stairs						30.028	.000
Help with other people		14	8.3%	26	37.7%		
Independent		154	91.7%	43	62.3%		
		**Mean**	**(SD)**	**Mean**	**(SD)**	**t**	**p-value**
Demographic Variables							
Age		76.53	(7.10)	78.57	(7.90)	-1.944	.053
Years of education		4.66	(4.60)	3.46	(3.21)	2.200	.029
No. of siblings alive		2.55	(2.67)	1.64	(2.69)	2.351	.020
No. of children alive		2.85	(1.82)	2.41	(1.86)	1.668	.097
No. of chronic diseases suffered		2.06	(1.48)	2.51	(1.54)	-2.071	.039
No. of leisure activities engaged in		1.99	(1.06)	1.48	(.75)	4.070	.000
Control variables							
LSNS - family network		9.98	(6.41)	6.67	(5.70)	3.735	.000
LSNS - friend network		9.10	(5.53)	6.93	(5.03)	2.818	.005
Life events (6 Items)		.18	(.39)	.41	(.65)	-2.682	.009
BI		98.63	(3.39)	93.20	(10.50)	4.207	.000
IADL		22.98	(3.36)	20.22	(4.66)	4.475	.000
Outcome measures							
LSI-A		14.64	(2.15)	8.46	(2.82)	16.377	.000
GDS		1.89	(1.75)	6.68	(2.87)	-12.917	.000
RSES		8.52	(1.42)	5.62	(2.17)	10.244	.000
GHS		8.07	(2.60)	13.62	(5.35)	-8.214	.000

Note :1. LSI-A = Life Satisfaction Scale, GDS = Geriatric Depression Scale, RSES = Rosenberg’s Self-Esteem Scale, GHQ = General Health Questionnaire, BI = Modified Barthel Index, IADL = Lawton Instrumental Activities of Daily Living Scale, LSNS = Lubben Social Network Scale.

2. *Multiple-response items between clusters were tested with two-proportion z-test.

Those in the active ageing cluster were more likely to be younger (mean age: 76.53 (AG) versus 78.57 (IG)), healthier with fewer chronic diseases (mean number of chronic diseases: 2.06 (AG) versus 2.51 (IG)), less depressed (mean scores: 1.89 (AG) versus 6.68 (IG)), more satisfied with their life (mean scores: 14.64 (AG) versus 8.46 (IG)), and to have higher self-esteem (mean scores: 8.52 (AG) versus 5.62 (IG)). They also enjoyed better sleep quality (less than one sleepless night per week: 71.69% (AG) versus 41.79% (IG)). Most of their income came from the support of their family members (60.84% (AG) versus 30.43% (IG)) and they were more satisfied with their economic condition (enough money for daily expenses: 41.87% (AG) versus 19.12% (IG)). They joined more leisure activities (average number of leisure activities engaged in: 1.99 (AG) versus 1.48 (IG)) and were more likely to have the ability to move freely (climbing stairs without help: 91.72% (AG) versus 62.32% (IG)). Furthermore, the subjects in this cluster were more likely to make friends with others (mean scores: 9.10 (AG) versus 6.93 (IG)), more willing to participate in group activities such as dancing, singing, and playing musical instruments for leisure (26.34% (AG) versus 9.09% (IG)). Their relationships with other family members were also better (mean scores: 9.98 (AG) versus 6.67 (IG)) and they met their family members more frequently (average number of meetings per month: 5.56 (AG) versus 3.84 (IG) (Table 2).

To develop a model that can be used to determine the characteristics that differentiate two groups, a discriminant analysis was performed. A total of 238 cases were randomly split into two sets of data, D1 and D2, which contained 109 and 129 candidates respectively. D1 was the test sample and D2 was the holdout sample, which served as internal validation. We first conducted the discriminant analysis on the test sample, and all of the variables found to be significantly different between the AG and IG were entered into the model. As a result, variables including GDS, GHQ, LSI-A, RSES, Life Event (LE), the Lubben Social Network Scale (LSNS), the Modified Barthel Index (BI), the Lawton Instrumental Activities of Daily Living Scale (IADL), the number of leisure activities engaged in, normal vision (including with corrective eyewear), income source, and sleep quality, were retained in the model. Discriminating power was evaluated by several criteria: (a) Wilks’ lambda, (b) variance explained, and (c) percentage correctly classified. The results are shown in Table 3. The discrimination model gave Wilks’ lambda = 0.28, Chi-square = 118.92, df = 13, and p <0.001, implying that the AG and IG clusters were significantly different with respect to the given discriminator variables in the model. In addition, the means of these variables for the two groups were also significantly different at the 5% level. Seventy-two per cent of the variation between the two groups was accounted for by these discriminating variables. Of these variables, LSI-A and GDS were the most significant factors contributing to the discriminant function. The resultant equation was then used to allocate the seniors to an appropriate group according to the seniors’ information in the D2 dataset. As a result, 98.51% and 94.12% of those in the AG and IG groups, respectively, could be correctly predicted, indicating that the model has a high level of predictive power.

**Table 3:** **Standardized canonical discriminant function coefficients**

Variables	Discriminant coefficients	Correlation with discriminant function
**Demographic variables**		
Normal vision (with corrective eyewear)		
- (Yes versus No)	.182	-.157
Income source		
- Government vs from family/own savings	.158	.174
Sleepless night		
- <1 per week vs at least once per week	-.124	-.300
Number of leisure activities engaged in	-.028	-.123
**Control variables**		
Lawton Instrumental Activities of Daily Living Scale (IADL)	-.186	-.206
Lubben Social Network Scale (LSNS) - Family subscale	.185	-.153
Life events (LE)	.142	.216
Lubben Social Network Scale (LSNS) - Friend subscale	-.047	-.181
Modified Barthel Index (BI)	.025	-.237
**Outcome measures**		
Life Satisfaction Scale (LSI-A)	-.699	-.737
Geriatric Depression Scale (GDS)	.535	.653
General Health Questionnaire (GHS)	.267	.385
Rosenberg’s Self-Esteem Scale (RSES)	.098	-.515

Note: All variables are significant at p = 0.05.

## Discussion

Those in the active ageing cluster were more likely to be younger and healthier, with fewer chronic diseases and with fewer depressive symptoms than those in the inactive ageing cluster. They were more satisfied with their lives and had higher self-esteem and better sleep quality. They were more satisfied with their financial situation (had enough money for daily expenses), and most of their income came from their family members. Their relationships with family members were also better. They met with their family members more frequently. They engaged in more leisure activities and were more likely to have the ability to move freely (climbing stairs without help) and were more willing to participate in group activities such as dancing, singing, and playing musical instruments in their leisure time.

Our findings are fairly similar to those reported by López et al. in a study that we located outside of the systematic literature search reported above [26]. López et al. wanted to establish the health and socio-cultural determinants of active ageing in a sample of 456 community living adults aged 54 to 75 years old in Spain. They found more active agers in men than in women, whereas gender was not a significant variable in our results. In their logistic regression model, being a woman and the number of diagnosed diseases were risk factors against active ageing, whereas years of education was a protective factor against the absence of active ageing. Similarly, our study showed that the number of chronic diseases and the number of years of education were significantly different in the AG and IG.

Because the sample consisted of participants who were social service centre members and likely to be more active than those who were not, the findings have to be interpreted with caution in view of the sampling bias. The WHO policy framework paper on active ageing advocates for the adoption of policies by governments to meet the challenges of global ageing [1]. Active ageing is not merely for senior citizens, but rather for people to realize their potential for health throughout the life course.

## Conclusion

Our findings add to the current limited knowledge about the correlates of healthy active ageing. Active ageing was observed in people with better health, and functional performance, as well as a more satisfying social network and relationships. Physical health, mental wellbeing, a good relationship with one’s family and a willingness to join in social and group activities – these dimensions in a person’s life are aspects that cannot be easily modified within the duration of a programmed intervention. As such, the promotion of active ageing must occur early in older age, or the sooner the better.
